# A Patient With Hepatocellular Carcinoma and Lung Metastasis Successfully Underwent Curative Surgery Following the Downstaging Treatment: A Case Report

**DOI:** 10.7759/cureus.64621

**Published:** 2024-07-15

**Authors:** Shiguo Xu, Ke Ma, Jianfeng Lu, Tao Wei, Risheng Que

**Affiliations:** 1 Department of Hepatobiliary and Pancreatic Surgery, The First Affiliated Hospital, Zhejiang University School of Medicine, Hangzhou, CHN

**Keywords:** pd-1 inhibitors, immunotherapy, transformative surgery, transarterial chemoembolization (tace), hepatocellular carcinoma

## Abstract

Hepatocellular carcinoma (HCC) is the most common primary liver malignancy. Hepatic resection constitutes the major curative treatment option, but a significant proportion of patients are not surgical candidates on initial evaluation. Along with the development of novel therapeutic strategies including targeted therapies and immunotherapies, a few HCCs can achieve tumor downstaging and be curatively resected. A 52-year-old man was diagnosed with HCC with portal vein invasion and extensive pulmonary and lymph node metastasis. Transarterial chemoembolization (TACE) in conjunction with donafenib and sintilimab was given. Primary tumors in the liver largely shrank with almost complete elimination of the lung metastases following treatment. The patient subsequently underwent curative surgery for HCC, and the pathological examination revealed complete necrosis of the tumor. Targeted immunotherapy was continued after surgery and no disease progression was found on the latest follow-up. Advanced HCC with distant metastasis might have an excellent response to combination therapy of TACE with tyrosine kinase-targeted inhibitors and PD-1 blocker, and achieve opportunity for curative surgery. This efficacy may be associated with the remodeling of immune microenvironment and angiogenesis. HCC is extremely heterogeneous, and the response to therapeutics varies among patients. There is a lack of useful biomarkers to predict therapeutic efficacy, which needs further studies.

## Introduction

Hepatocellular carcinoma (HCC) is the most common primary liver cancer. The most effective treatment for HCC is radical resection, but a considerable proportion of patients cannot undergo curative resection at the time of initial diagnosis. For unresectable HCC, other therapeutic strategies including interventional therapy, radiotherapy, and chemotherapy can be employed to facilitate tumor downstaging [1]. Traditional methods of transformation include transarterial chemoembolization (TACE) and radiofrequency ablation (RFA). In recent years, immunotherapy and molecular-targeted therapy technologies have made great progress and have been widely used in the transformative treatment of patients with end-stage HCC, achieving considerable transformation rates [2].

Donafenib is a novel small molecule multi-target tyrosine kinase inhibitor (TKI) that inhibits the activity of multiple tyrosine kinase receptors, such as vascular endothelial growth factor receptor (VEGFR) and platelet-derived growth factor receptor (PDGFR), thereby inhibiting tumor cell proliferation and angiogenesis [3]. As a deuterated derivative of sorafenib, donafenib exhibits a reduced incidence of drug-related adverse events due to its enhanced structural stability [4]. In a phase II-III clinical trial, donafenib demonstrated superior efficacy to sorafenib in improving overall survival (OS) in Chinese patients with advanced HCC and exhibited a favorable safety and tolerability profile. It has been approved as a standard targeted therapy for patients with advanced HCC [5].

Sintilimab is a highly selective programmed cell death protein 1 (PD-1) immune checkpoint inhibitor (ICI), which can block the PD-1/PD-L1 signaling pathway, thereby activating the immune system and enhancing its ability to recognize and kill tumor cells. Although ICI therapy has been widely used in the treatment of patients with advanced HCC, several clinical studies have demonstrated that PD-1 inhibitor monotherapy cannot provide ideal therapeutic effects [6]. Therefore, the combination of ICI and TKI is necessary. TKI can regulate the tumor immune microenvironment and enhance the anti-tumor effect of ICI [7]. A study has demonstrated that the combination of PD-1 inhibitors with anti-VEGF-targeted drugs shows better therapeutic effects than monotherapy in the systemic treatment of patients with advanced HCC [8].

The case study details a patient who received several TACE procedures and a combination of targeted immunotherapy with donafinil and sindilizumab resulting in partial remission of the lesion. Ultimately, the lesion was successfully resected through transformative surgery.

## Case presentation

A 52-year-old male was admitted to our hospital on June 26, 2023, for a routine physical examination that revealed hepatic occupancy. The patient had a history of hepatitis B infection but normal liver function. Laboratory examinations of the patient revealed that the alpha-fetoprotein (AFP) level was 71.9 ng/mL and protein induced by vitamin K absence or antagonist-II (PIVKA-Ⅱ) level was 25814 mAU/mL. Enhanced magnetic resonance imaging (MRI) and enhanced computed tomography (CT) of the liver suggested that a giant HCC of the right liver with a diameter of approximately 13 cm was accompanied by multiple stellate lesions, involving the right hepatic vein and the right branch of the portal artery, and accompanied by multiple cancerous thrombus formation in the right branch of the portal artery. Multiple enlarged lymph nodes at the retroperitoneal hilar and adjacent to the abdominal aorta were considered as metastatic foci. Lung CT showed multiple nodules in both lungs, metastatic tumor considered. Positron emission tomography-computed tomography scan (PET-CT) of the whole body showed a huge uneven density mass (maximal diameter about 13 cm) in the right lobe of the liver, with unevenly increased FDG metabolism, considering HCC with necrosis and hemorrhage, and involvement of the right branch of the portal vein; multiple solid nodules in both lungs, pleura, and interlobular subpleura, with a slight increased FDG metabolism, considered multiple metastatic tumors; multiple slightly larger lymph nodes in both clavicular regions, right heart diaphragm angle, porta hepatis, hepatic-gastric hiatus and retroperitoneum, with a slight increased FDG metabolism, considered possible lymph node metastasis. Combined with the patient's imaging, tumor marker results, and medical history, he was diagnosed with HCC with distant metastasis, which was not suitable for surgical resection. A comprehensive treatment plan of TACE combined with targeted immune drugs was then developed.

The patient underwent a total of four TACE (oxaliplatin 75 mg, raltitrexed 2 mg, and idarubicin 10 mg) in combination with bronchial arterial infusion (BAI) (oxaliplatin 75 mg, raltitrexed 2 mg). Two weeks after diagnosis, the patient started the targeted therapy of donafenib, and received four times of sindilizumab immunotherapy in sequence. Following four months of treatment with regular rechecks, a liver CTA revealed a right liver cancer lesion measuring approximately 8.2 × 8.0 cm. This was a notable reduction from the lesion four months prior. Additionally, a lung CT angiography (CTA) demonstrated a reduction in the number of metastases in both lungs compared to previous lesions.

**Figure 1:**
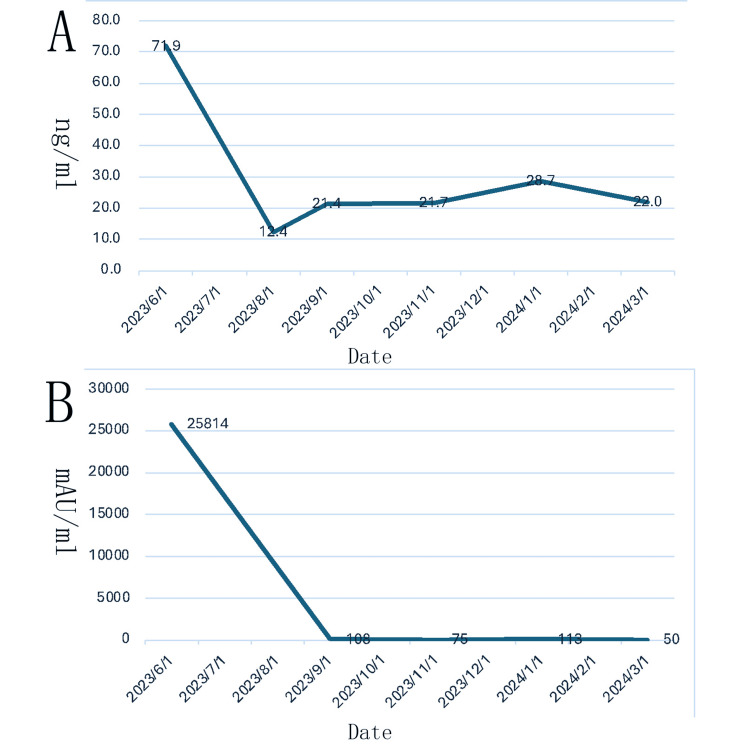
After treatment with donafenib in combination with sindilizumab, the patient's AFP (ng/mL) and PIVKA-Ⅱ (mAU/mL) levels decreased significantly. A. Dynamics of AFP (ng/ml) levels after treatment; B. Dynamics of PIVKA-Ⅱ (mAU/ml) levels after treatment. AFP: alpha-fetoprotein; PIVKA-II: protein induced by vitamin K absence or antagonist-II

**Figure 2:**
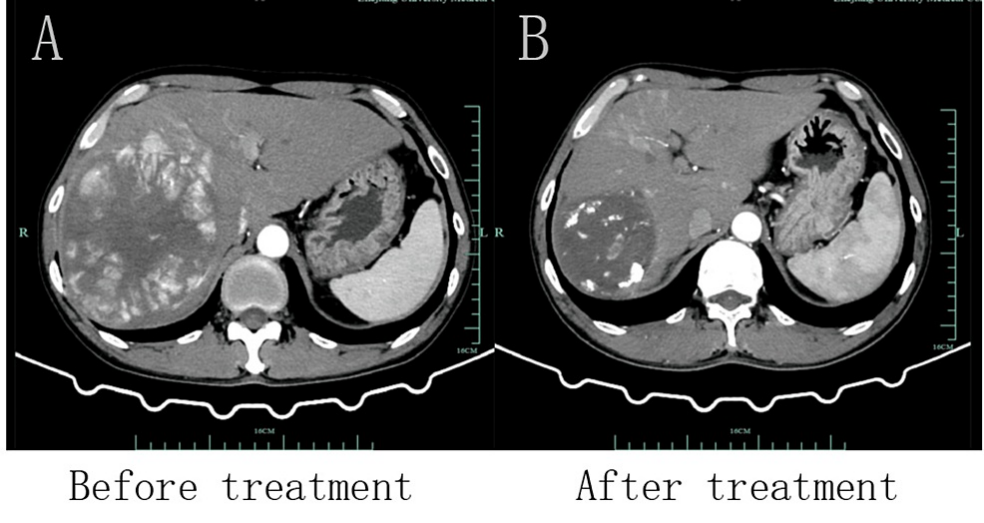
After TACE treatment with targeted immunotherapy, the patient's liver lesions reduced significantly. A. CTA image of the liver before treatment; B. CTA image of the liver after treatment. TACE: transarterial chemoembolization; CTA: computed tomography angiography

**Figure 3:**
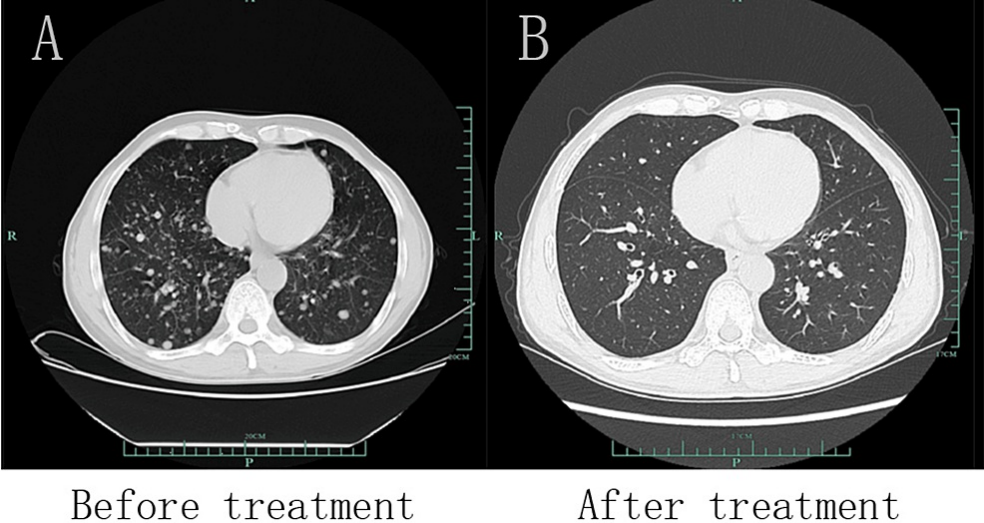
After TACE treatment with targeted immunotherapy, the metastatic lesions in the lungs had almost completely disappeared, with only one remaining in the left lung. A. CT image of the lung before treatment; B. CT image of the lung after treatment. TACE: transarterial chemoembolization

**Figure 4:**
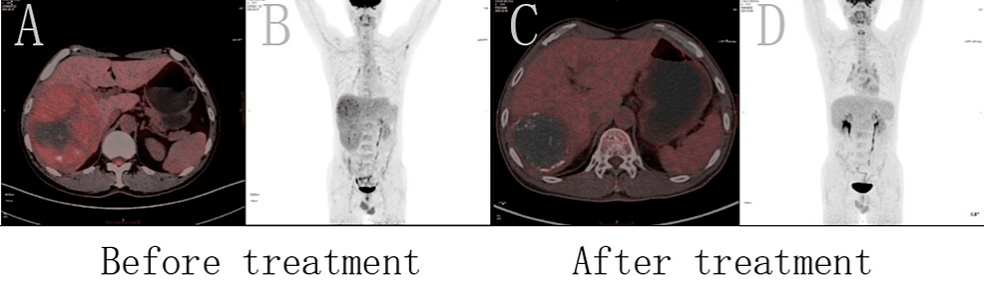
After TACE treatment with targeted immunotherapy, the patient's lesions were significantly smaller than before with decreased metabolism. A. Fusion image of PET-CT before treatment; B. MIP image before treatment; C. Fusion image of PET-CT after treatment; D. MIP image after treatment. TACE: transarterial chemoembolization; PET-CT: positron emission tomography-computed tomography scan; MIP: maximum intensity projection

The patient was readmitted to the hospital after eight months of treatment, and laboratory examinations showed that AFP was 22.0 ng/mL and PIVKA-Ⅱ was 50 mAU/mL, both of which had decreased significantly compared to the pre-treatment period (Figure 1). Liver CTA shows that after TACE for HCC with multiple stellate lesions, the lesions are significantly smaller than before (Figure 2). Lung CT shows almost total disappearance of metastatic lesions in the lungs, with only one remaining in the left lung (Figure 3). PET-CT of the whole body showed post-comprehensive treatment status of HCC and multiple flaky, round-like mass shadows were observed in the liver, especially in the right liver, accompanied by iodine oil deposition. In comparison to the previous PET-CT, the lesions were significantly smaller, with decreased metabolism (Figure 4); multiple small nodules in both lungs, with significantly fewer and smaller lesions than before, and decreased metabolism.

**Figure 5:**
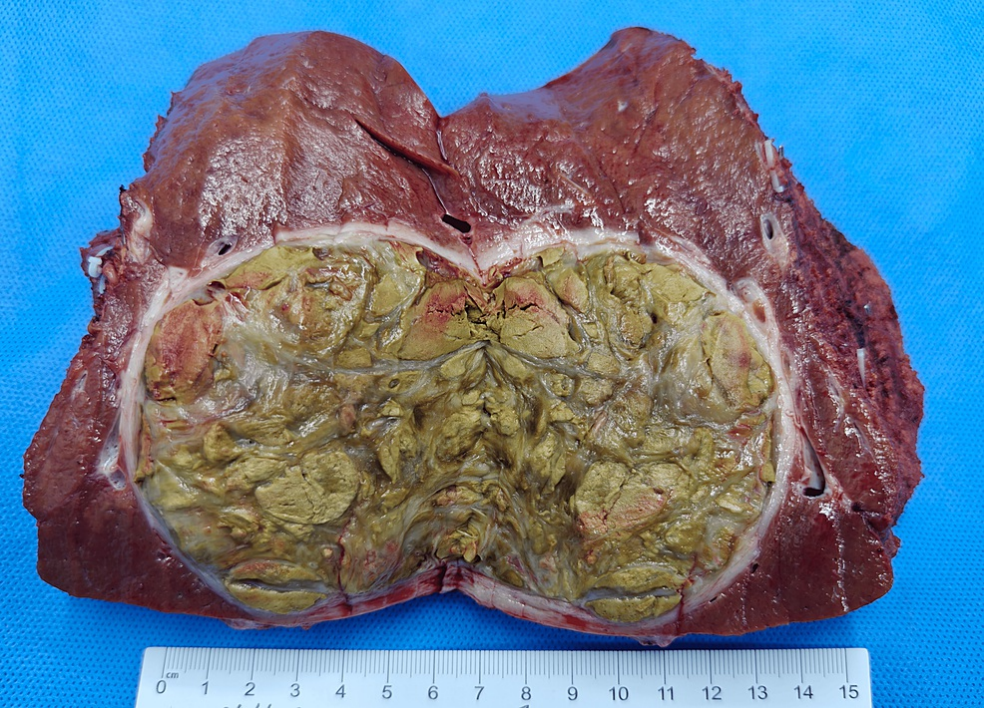
Surgical specimen of HCC removed from the patient. HCC: hepatocellular carcinoma

After a multidisciplinary team (MDT) discussion, right hemihepatectomy was recommended, postoperative targeted immunotherapy was continued, and stereotactic body radiation therapy (SBRT) was feasible for lung nodules. The patient underwent a complex hepatectomy for HCC under general anesthesia on March 19, 2024. Intraoperative shows obvious nodular cirrhosis of the liver and a huge tumor measuring approximately 8.0 × 8.0 cm in the right liver. Postoperative pathology shows complete necrotic nodule (8 × 7.5 × 6.5 cm) with fibrous tissue proliferation, small bile duct hyperplasia, histiocyte aggregation, chronic inflammatory cell infiltration, and focal multinucleated giant cell reaction. No clear evidence of residual cancerous tissue was observed, which is consistent with the changes associated with HCC following TACE, and no evidence of cholangiocarcinoma embolus or nerve involvement (Figure 5). The patient recovered well 10 days after surgery and was discharged from the hospital with continued targeted immunotherapy.

## Discussion

Clinical guidelines for patients with advanced HCC recommend systemic therapy. With the advances in treatment in recent years, new targeted therapeutic agents and immunotherapies have been widely used. Studies have shown that the combination of the two has a certain transformation rate. In a phase Ib study evaluating the safety of lenvatinib in combination with pembrolizumab in patients with unresectable HCC, no new adverse events were identified and the partial response (PR) rate was 46%. In another phase I study combining camrelizumab and apatinib in patients with advanced HCC, the PR rate was 50%. Patients who underwent conversion surgery following comprehensive treatment exhibited a significantly improved prognosis, even in the presence of extrahepatic metastases [9].

In current transformative therapeutic regimens, combination therapy with multikinase inhibitors and ICIs has shown favorable efficacy. In an IMBrave150 trial, the combination of bevacizumab (anti-VEGF-A antibody) and atezolizumab (anti-PD-L1 antibody) showed better OS than sorafenib, the standard of care for first-line systemic therapy in metastatic HCC [10]. In addition, the results of the phase I/II trial of the COSMIC-312 trial showed that the combination of cabozantinib, a multikinase inhibitor with VEGF-inhibitory activity, and atezolizumab had favorable therapeutic effects in metastatic HCC [11].

Studies have demonstrated that VEGF binding to its receptor can inhibit the maturation and antigen presentation process of dendritic cells, and simultaneously induce the expression of PD-L1 receptor on the surface of the cells, thereby inhibiting the killing effect of the immune system on tumor cells [12]. Furthermore, elevated levels of VEGF result in the inhibition of the proliferation and cytotoxic capacity of T cells [13]. These suggest that VEGF plays an important role in tumor microenvironment (TME) formation and tumor immune escape. Therefore, the use of multikinase inhibitors that encompass VEGF, such as donafenib, can inhibit tumor growth not only by inhibiting angiogenesis but also by immune-related mechanisms. However, the use of VEGF inhibitors alone is not sufficient to generate a complete immune response against tumors. In a clinical trial of patients with solid tumors treated with the VEGF inhibitor aflibercept, antigen-specific immune responses did not improve despite an increase in the number of mature dendritic cells [14]. Therefore, the combination of anti-angiogenic drugs with immunotherapy is recommended to enhance the immune-killing effect on tumors.

In addition, studies have demonstrated that immunotherapeutic agents can enhance the efficacy of anti-angiogenic drugs. This effect may be related to the increased secretion of interferon-gamma (IFNγ) by activated CD8+ T cells in response to immunotherapy [15]. It has been demonstrated that IFNγ is capable of downregulating the expression of delta-like protein 4 (dLL4) on endothelial cells, which in turn inhibits Notch signaling required for angiogenesis [16]. Meanwhile, IFNγ can also suppress VEGF mRNA transcript levels in endothelial cells, thereby exerting an anti-tumor angiogenesis effect [17]. Therefore, immunotherapeutic drugs can facilitate vascular remodeling by activating tumor-specific immune cells and promoting the secretion of IFNγ. Concurrently, this effect can also promote the aggregation of immune cells at the tumor site. This may be one of the relevant mechanisms of targeted combined immunotherapy for HCC, although the specific mechanism involved remains to be further elucidated. In this study, the surgical specimens could not be retained with active tissue due to total necrosis. Furthermore, no tissue puncture was performed prior to treatment to allow further characterization of gene variation and expression. This represents a significant limitation of the study.

There have been many previous case reports of targeted immunotherapy for advanced HCC followed by transformative surgery. However, the majority of these reports have employed either atezolizumab in combination with bevacizumab or lenvatinib in combination with camrelizumab. These reports strongly suggest that the combination of anti-angiogenic therapy with IHI therapy can induce a strong systemic antitumor response, providing a feasible idea for the treatment of end-stage HCC. And the combination of donafenib and sindilizumab, a TKI with VEGF inhibitory activity, should also have comparable therapeutic effects.

## Conclusions

In the present study, we report the case of a significant reduction in primary lesion size and intrapulmonary metastases following multiple TACE procedures in conjunction with donafenib and sindilizumab, ultimately resulting in successful transformative surgery. It can be inferred that current combination therapies may have significant efficacy in some patients with advanced HCC with extensive systemic metastases, and further studies are needed to explore the mechanisms of treatment response and effective means of predicting efficacy.
